# Mechanism Study of Pulsus Paradoxus Using Mechanical Models

**DOI:** 10.1371/journal.pone.0057512

**Published:** 2013-02-28

**Authors:** Chang-yang Xing, Tie-sheng Cao, Li-jun Yuan, Zhen Wang, Kun Wang, Hua-ri Ren, Yong Yang, Yun-you Duan

**Affiliations:** Department of Ultrasound Diagnostics, Tangdu Hospital, Fourth Military Medical University, Xi’an, China; University of Leicester, United Kingdom

## Abstract

Pulsus paradoxus is an exaggeration of the normal inspiratory decrease in systolic blood pressure. Despite a century of attempts to explain this sign consensus is still lacking. To solve the controversy and reveal the exact mechanism, we reexamined the characteristic anatomic arrangement of the circulation system in the chest and designed these mechanical models based on related hydromechanic principles. Model 1 was designed to observe the primary influence of respiratory intrathoracic pressure change (RIPC) on systemic and pulmonary venous return systems (SVR and PVR) respectively. Model 2, as an equivalent mechanical model of septal swing, was to study the secondary influence of RIPC on the motion of the interventriclar septum (IVS), which might be the direct cause for pulsus paradoxus. Model 1 demonstrated that the simulated RIPC had different influence on the simulated SVR and PVR. It increased the volume of the simulated right ventricle (SRV) when the internal pressure was kept constant (8.16 cmH_2_O), while it had the opposite effect on PVR. Model 2 revealed the three major factors determining the respiratory displacement of IVS in normal and different pathophysiological conditions: the magnitude of RIPC, the pressure difference between the two ventricles and the intrapericardial pressure. Our models demonstrate that the different anatomical arrangement of the two venous return systems leads to a different effect of RIPC on right and left ventricles, and thus a pressure gradient across IVS that tends to shift IVS left- and rightwards. When the leftward displacement of IVS reaches a considerable amplitude in some pathologic condition such as cardiac tamponade, the pulsus paradoxus occurs.

## Introduction

Pulsus paradoxus is a condition whereby normal inspiratory causes a fall of systolic blood pressure of greater than 10 mmHg [1]. It was first described by Kussmaul in constrictive pericarditis, and then found more commonly associated with pericardial tamponade and also with other clinical conditions [2].

There have been many studies investigating the mechanism of pulsus paradoxus. Some studies proposed that an inspiratory increase in right ventricular filling precedes a decrease in left ventricular filling due to the increased pulmonary venous compliance [3], [4], which was called “lung pooling” hypothesis, i.e. series ventricular interdependence. Other studies observed an inverse relation in left and right ventricular ejection dynamics that was very close to 180° out of phase and a septal swing with the dynamics, i.e. parallel ventricular interdependence [5]–[8]. The experimental study by Gonzalez et al [9] and our experimental and clinical studies [10]–[12] do not support “lung pooling” hypothesis, but could be easily explained by the parallel ventricular interdependence. Although the clinical manifestations of the dynamics of 180° out of phase and a septal swing that described by Gonzalez and our studies were commonly observed and accepted, the underlying mechanical principle is still unclear.

Based on all the published data of pulsus paradoxus and our studies on the mechanism, we hypothesized that the characteristic anatomical arrangement of the circulation system in the chest cavity is the key to the swing of the ventricular septum. In our hypothesis, the respiratory intrathoracic pressure change (RIPC) is applied through this structure on the left side of the septum and drives it swing. The septal swing changes the short axis of the two ventricles in an inverse relation that alternates the left and right ventricular ejection dynamics in about 180° out of phase. We named this characteristic anatomic structure the “pressure amplifier in the chest”, or simply, the “amplifier”, because it may amplify the RIPC of about 4 mmHg (the input of the amplifier) to a respiratory blood pressure variation of 10 mmHg normally, and in the typical condition of cardiac tamponade, it may amplify the same intrathoracic pressure change to a respiratory blood pressure variation (the output of the amplifier) up to almost 100 mmHg [13], [14].

To prove the proposed pressure amplifier mechanism for pulsus paradoxus and to demonstrate that the septal swing is driven by the RIPC, we simulated the characteristic anatomical arrangement of the circulation system using in vitro models based on related hydromechanic principles.

## Materials and Methods

### 1. Hemodynamic Analysis of the Circulation System

**Figure 1:**
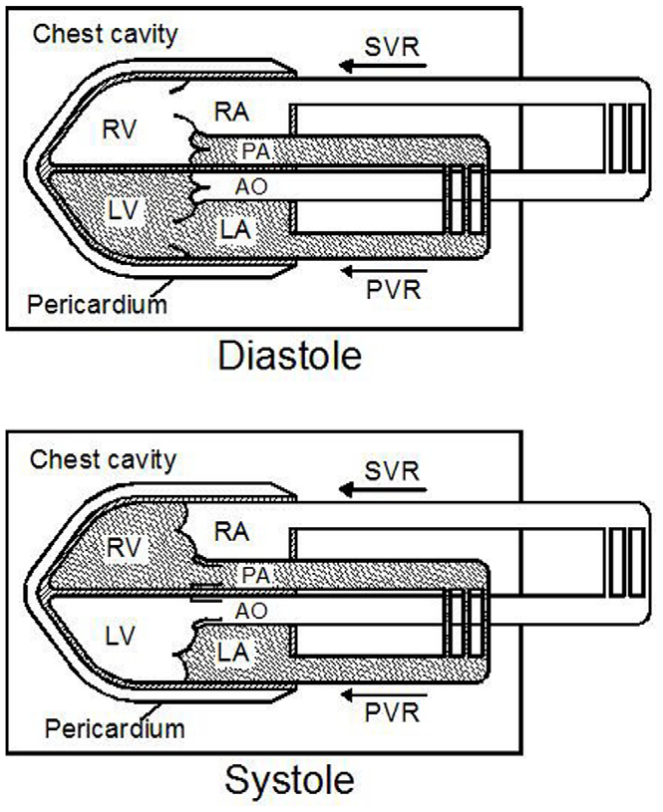
Hemodynamic analysis of the circulation system. The circulation system may hydromechanically be divided into two enclosed fluids, the WITS and PITS (the white and the dark parts of the circulation system in the figure). They are operating at a very different pressure level and kept separated by either the atrio-ventricular valves in systole (top panel) or the semilunar valves in diastole (bottom panel), though they are connected in series. The systemic venous return (SVR) system consists of systemic vasculature and right heart, and the pulmonary venous return (PVR) system consists of pulmonary vasculature and left heart. Their location relative to WITS and PITS changes in diastole and systole. Modified from Wang et al. *PLoS ONE.* [10].

Functionally, the circulation is divided into systemic and pulmonary circulation systems. While from the hemodynamic point of view, we propose to divide the whole circulation system into two closed portions which are separated by the semilunar valves in diastole and by the atrio-ventricular valves in systole (Figure 1).

One portion (the dark part in the upper panel of Figure 1) is from the pulmonary valve, through the pulmonary artery, pulmonary vascular bed, pulmonary vein, left atrium, mitral valve, left ventricle to the aortic valve. In diastole, this portion is within a hermetically-sealed chamber, the chest cavity. We thus call this portion of the circulation the whole-intrathoracic system (WITS). In contrast to WITS, the other portion of the circulation (the white part in the upper panel of Figure 1) is not completely within the chest cavity and we thus call it the partially-intrathoracic system (PITS).

Though there is a periodic blood flow within and intermittent blood exchange between the two systems, they normally keep separated from each other by closed valves, either semilunar (diastole) or atrio-ventricular (systole). Although these two portions of the circulation are connected in series, their internal pressure levels are relatively independent, i.e., their arterial pressure may be respectively increased and their ventricular filling pressure may be respectively elevated without an obvious or direct influence one another. The respiratory intrathoracic pressure change (RIPC) is the force exerted externally on WITS and the intrathoracic part of PITS. Whereas, the myocardium contraction or the tension of the walls of the cardiovascular system is the driven force to maintain the blood circulation and the pressure in it. WITS is directly exposed to RIPC, except for the areas of interatrial and interventricular septa and the cardiac valves which are indirectly exposed to the atmospheric pressure through a part of PITS (Figure 1).

For the venous return of the circulation, we also separate it into two systems. One may be called the pulmonary venous return system (PVR) and the other, the systemic venous return system (SVR) (Figure 1). The peripheral ends of the two systems are capillary vascular beds, the central ends of the systems are left (PVR) or right heart (SVR). During diastole, PVR is a part of WITS, and SVR is a part of PITS. Although in systole this division is partly exchanged, it does not interfere with our hemodynamic analysis. The peripheral end of the SVR is outside the chest cavity, the central end of SVR and the whole PVR are within the chest cavity.

We have found through our study series [10]–[12] that this seemingly small difference in the anatomic arrangement between these two circulation systems may lead to the pulsus paradoxus in various pathological conditions.

### 2. Simulated Models of the above Circulation Systems

To analyze the effects of simulated RIPC on the above two different systems, we designed two models.

#### 2.1. Model 1 and tests for observing the influence of RIPC on SVR and PVR separately

**Figure 2:**
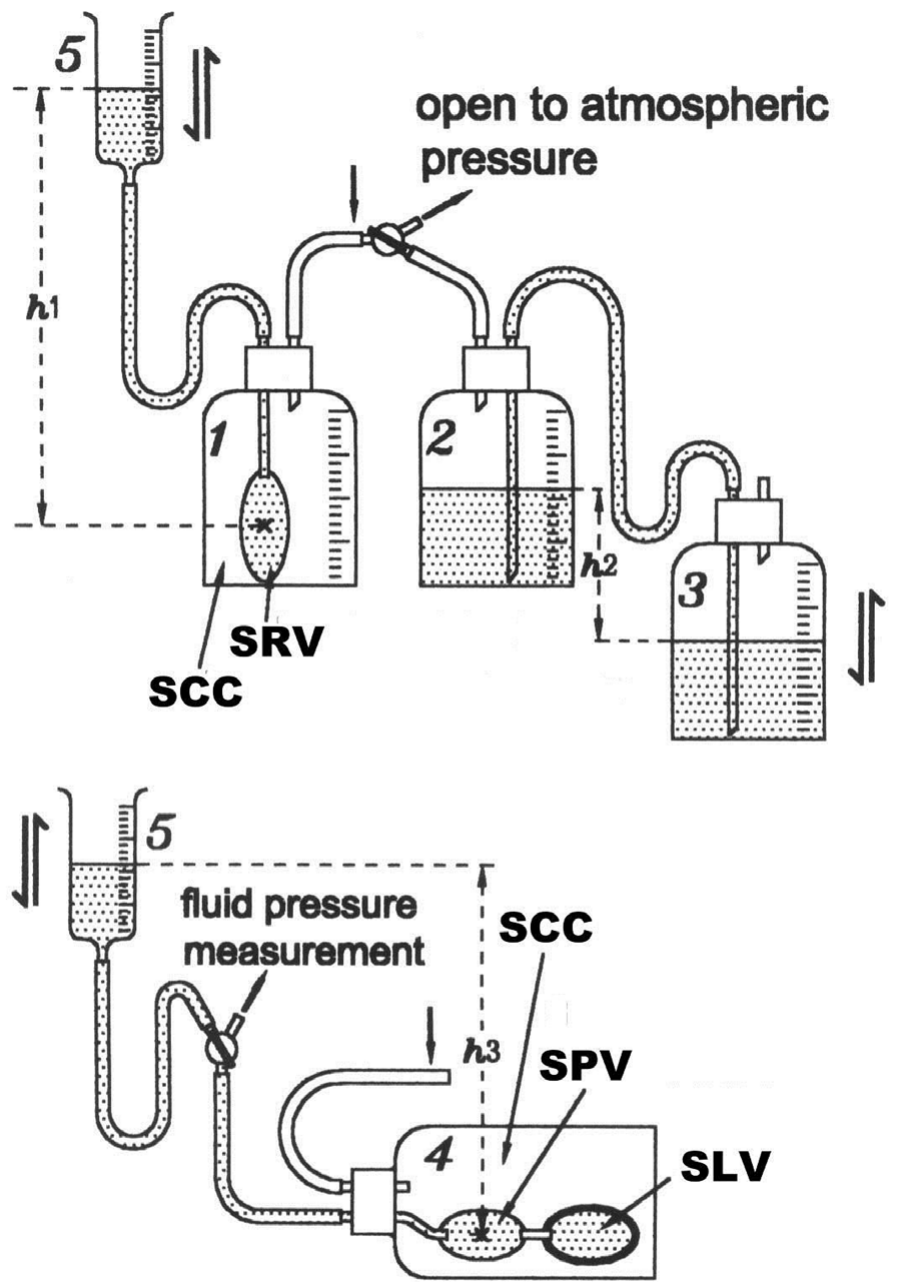
Scheme of Model 1. Five bottles labeled with number 1 to 5 were used and were connected by rubber and glass tubing. No. 5 is a syringe of 50 C.C. without the piston. The top panel is a simulated SVR connected with a negative-positive-pressure-generator (Bottle No.2 and 3) through rubber and glass tubing. The bottom panel is the simulated PVR. SCC = simulated chest cavity, SLV = simulated left ventricle, SRV = simulated right ventricle, SVR = systemic venous return system, PVR = pulmonary venous return system.

To observe the direct hydromechanical effects of the simulated RIPC on the SVR and PVR, respectively, we designed the mechanical Model 1 (Figure 2).

In this model, the simulated SVR was shown on the top panel of figure 2. Bottle 1 was a simulated chest cavity (SCC) with a simulated right ventricle (SRV) in it. SRV was made of a rubber balloon with 0.17 mm wall thickness which is connected with a syringe without the piston labeled number 5 (No.5) by the rubber and glass tubing. Bottle No.5 is simulated peripheral vascular bed providing an adjustable static pressure in this simulated SVR. The height from the fluid level in No.5 to the central point of SRV, the h_1_, is the filling pressure of SRV and could be adjusted. Considering the incompressibility of the water, the volume change of SRV caused by the h_1_ change can be read directly from the scale on the No.5. Bottles No.2 and No.3 constitute a negative and positive pressure generator to create a pressure in the SCC above or below the atmospheric pressure. The fluid level difference between these two bottles, the h_2_, is the pressure value in cmH_2_O that the negative and positive pressure generator generates. The height of bottle No.3 is adjustable. When the fluid level in No.3 is lower than that in No.2, the pressure generator produces negative pressure in SCC and vice versa. The zero pressure of the negative and positive pressure generator should be calibrated before it is connected with a three-way valve. Zero pressure will be reached when the h_2_ equals zero. The pressure generator may create negative pressure in SCC and acts as simulated RIPC if it is connected with bottle No.1 or bottle No.4 at the position where the vertical arrow indicated in the figure.

The simulated SVR was shown on the bottom panel of figure 2, which consisted of a simulated chest cavity (bottle No.4), a simulated left ventricle (SLV) which was made of a rubber balloon with the wall thickness of 0.34 mm, and simulated pulmonary vasculature (SPV), which was made of a rubber balloon with a wall thickness of 0.17 mm and is connected with No.5 through a three-way valve. This part of the model was also connected with the negative and positive pressure generator at the point of the vertical arrow indicated in figure 2 (in this situation, the bottle No.1 would be replaced.).

Test 1 and 2 were for observing the influence of RIPC on SVR. Test 1 was to observe the effects of the filling pressure increase in SRV (the h_1_ increase) and the negative pressure increase in SCC (the h_2_ increase) on the volume change of SRV separately.

To simplify the experiment procedure, the unit of 4.08 cmH_2_O was used as an interval pressure change which equals 3 mmHg. Two methods of filling were designed. The first method to test RIPC on the simulated SVR was to change the SRV pressure. The relationship between the SRV volume change and the SRV pressure (h_1_) was observed. This was done by shifting the three-way valve to the atmospheric pressure. The volumes of the SRV were recorded in each pressure change. The baseline SRV filling pressure is 8.16 cm H_2_O (4.08×2 cmH_2_O), i.e., the original h_1_ equals 8.16 cmH_2_O, which serves to distend the SRV, and then the fluid level of No.5 was elevated four times, each time by 4.08 cm. Then the SRV pressure (h_1_) was reset to the baseline, 8.16 cmH_2_O. The second method to test RIPC on the simulated SVR was to change the simulated intrathoracic pressure (h_2_). With h_2_ equals zero the negative and positive pressure generator was connected with bottle No.1 through the three-way valve. We called this condition of the h_1_ at baseline (8.16 cm H_2_O) and the h_2_ at zero, the baseline condition of the simulated SVR. Then the bottle No.3 was moved down four steps to let h_2_ increase 4.08 cm (3 mmHg) each time from its zero point and the SRV volume was recorded.

Test 2 was to observe the effect of simultaneous change of filling pressure in SRV and the negative pressure in SCC on the volume change of SRV. With the bottle No.3 and bottle No.5 moving up and down synchronically at the same rate from the baseline condition of the simulated SVR, i.e., the h_1_ and the h_2_ changing correspondingly, the volume changes of the SRV were observed.

Test 3 was for observing the influence of RIPC on PVR. This test allowed us to observe if the simulated RIPC might fully be transmitted into the simulated system. Firstly, the three-way valve between the No.5 and the bottle No.4 was shifted to connect these two bottles to have the SPV and the SLV to be filled at baseline filling pressure (h_3_ equals 8.16 cm) and then, the three-way valve was shifted to connect bottle No.4 with the fluid pressure measurement instrument (Hellige EK36) to observe the pressure changes in the SPV and SLV following pressure change in the SCC.

#### 2.2. Model 2 and tests for observing the secondary influence of RIPC on the motion of IVS

Model 2 was an equivalent mechanical model of septal swing. It was used to observe if the interventricular septum (IVS) would swing left- and rightwards under a simulated RIPC and to find major factors determining the swing amplitude of IVS.

**Figure 3:**
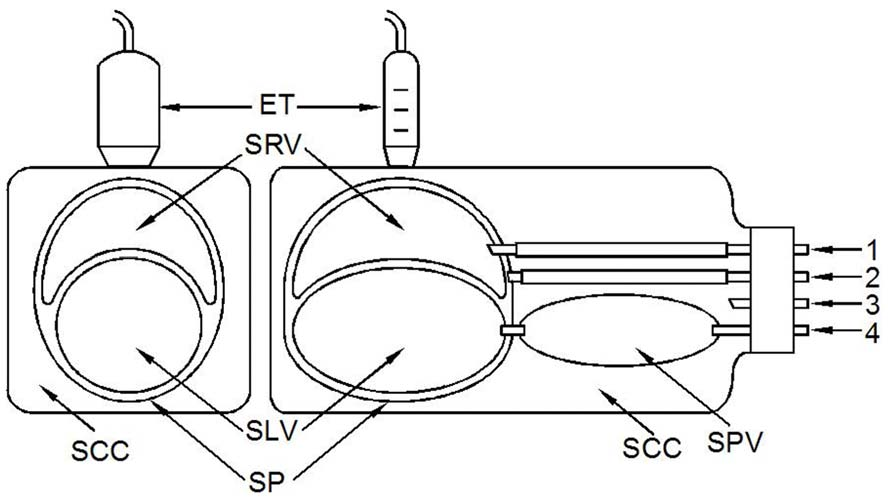
Scheme of Model 2. The right part of the figure is the longitudinal view of Model 2. The materials used are the same as those of Model 1. The top crescent structure is simulated right ventricle (SRV) and the bottom circular one is simulated left ventricle (SLV). Their common wall between SRV and SLV is the simulated interventricular septum. The thickness of the walls are 1.7mm, 3.4mm, 3.4mm from top to bottom in the figure. The left part is the short-axis view of model 2 which looks like a 2D-echocardiogram of the Model. The wall of these bottles are thin plastic and thus can be imaged by echocardiography.

This model was reformed from the combination of the two parts of Model 1. As figure 3 showed, the simulated right and left ventricles shared the common IVS and the pericardium. All the simulated cavities were filled with water and the pressures in them could be quantitatively adjusted through the connected tubing.

Acuson’s Sequoia 512 ultrasonographic system was used in this study to record the M-mode and two-dimensional cineloop of the Model 2.

Test 1 was to observe the effect of the simulated RIPC on the motion of IVS. Firstly, the pressures in SRV, SLV, SCC and simulated pericardium were adjusted to 4 mmHg, 9 mmHg, 0 mmHg and 0 mmHg respectively, which was called the standard pressure condition for model 2. Then the pressure in SCC was changed from –5 to +5 mmHg step by step with 1 mmHg (1.36 cmH_2_O) interval and the position and displacement of the simulated IVS was recorded and measured with the echocardiograph. Once the relationship of the pressure in SCC and the position of simulated IVS were determined, a rhythmic SCC pressure variation of 0 to −4 mmHg was applied to simulate the RIPC, the simulated IVS motion was observed and recorded with an echocardiograph.

Test 2 was to observe if the pressure difference between SRV and SLV may influence the displacement amplitude of the simulated IVS while the pressure variation in SCC, or the simulated RIPC, were kept unchanged. In this test the simulated RIPC was set at 0 to −4 mmHg at a rhythmic rate of 20 cycles per minute. The pressure in SLV was fixed at 9 mmHg and the pressure in SRV was set at 4, 5, 6, 7, 8 and 9 mmHg respectively.

Test 3 was to observe the effect of the pressure in pericardium on the motion of IVS. Firstly, the model was set at the standard pressure condition. Then the water was injected gradually through the tubing that connected with the simulated pericardial space. The size change of SRV and SLV was observed with echocardiograph. When the pressure in SRV was increased to be equal to that in SLV, 9 mmHg, the displacement amplitude of the simulated IVS with the simulated RIPC variation of 0 - −4mmHg was observed, respectively.

## Results

### 1. Result of Model 1

#### 1.1. The volume change of SRV with the two filling methods (Test 1 and 2)

For Test 1, the results of the SRV volume measurements with the two methods of filling were listed in Table 1. The baseline filling of h_1_ is 8.16 cm with the SCC under the atmospheric pressure and the baseline filling of h_2_ is zero with the h_1_ kept at 8.16 cm in all the five measurements.

**Table 1:** Comparison of the SRV volume changes during the adjustments of the SRV filling pressures and the simulated intrathoracic pressures respectively.

h_1_ (cm)	SRV volume (ml) with h_1_ change	h_2_ (cm)	SRV volume (ml) with h_2_ change
8.16(4.08×2)	78.5	0	78.6
12.24(4.08×3)	84.2	4.08	84.3
16.32(4.08×4)	90.0	8.16	90.1
20.40(4.08×5)	96.1	12.24	96.2
24.48(4.08×6)	102.8	16.32	102.6

SRV = simulated right ventricle; h_1_ = the SRV filling pressure; h_2_ = negative pressure generated by negative and positive pressure generator.

In Test 2, with the bottle No.3 and No.5 moved up and down synchronically, the SVR volume did not show any change, i.e., the RV volume kept constant when the internal and external RV pressures increased or decreased at the same time.

The results of these two tests indicated that the internal simulated right ventricular filling pressure and the externally applied simulated RIPC had equivalent effects on distending the SRV and that the externally applied simulated RIPC was fully transformed into the transmural distending pressure of SRV to increase the volume of SRV.

#### 1.2. The pressure change of SLV and SPV with the simulated RIPC (Test 3)

In test 3,with the baseline pressure of 8.16 cmH_2_O in the SLV and SPV, the negative and positive pressure generator produced a pressure range of positive pressure of 40.8 cmH_2_O (+30mmHg) to negative pressure of 40.8 cmH_2_O (−30mmHg). The pressure measured with the Hellige EK36 indicated the corresponding pressure of the h_3_ change, i.e., the pressure change produced by the negative and positive pressure generator was fully transmitted into the system, the SPV and the SLV. No SLV or SPV volume change was found during the SCC pressure changing, though their wall thickness were different.

The major differences of the influence of the simulated RIPC on the two simulated venous return systems were that the simulated RIPC altered the volume of SRV when the internal filling pressure was kept constant (8.16 cmH_2_O), while it changed only the static pressure in the simulated PVR system when its volume was kept constant.

### 2. Result of Model 2

#### 2.1. Effect of the simulated RIPC on the motion of the simulated IVS

**Figure 4:**
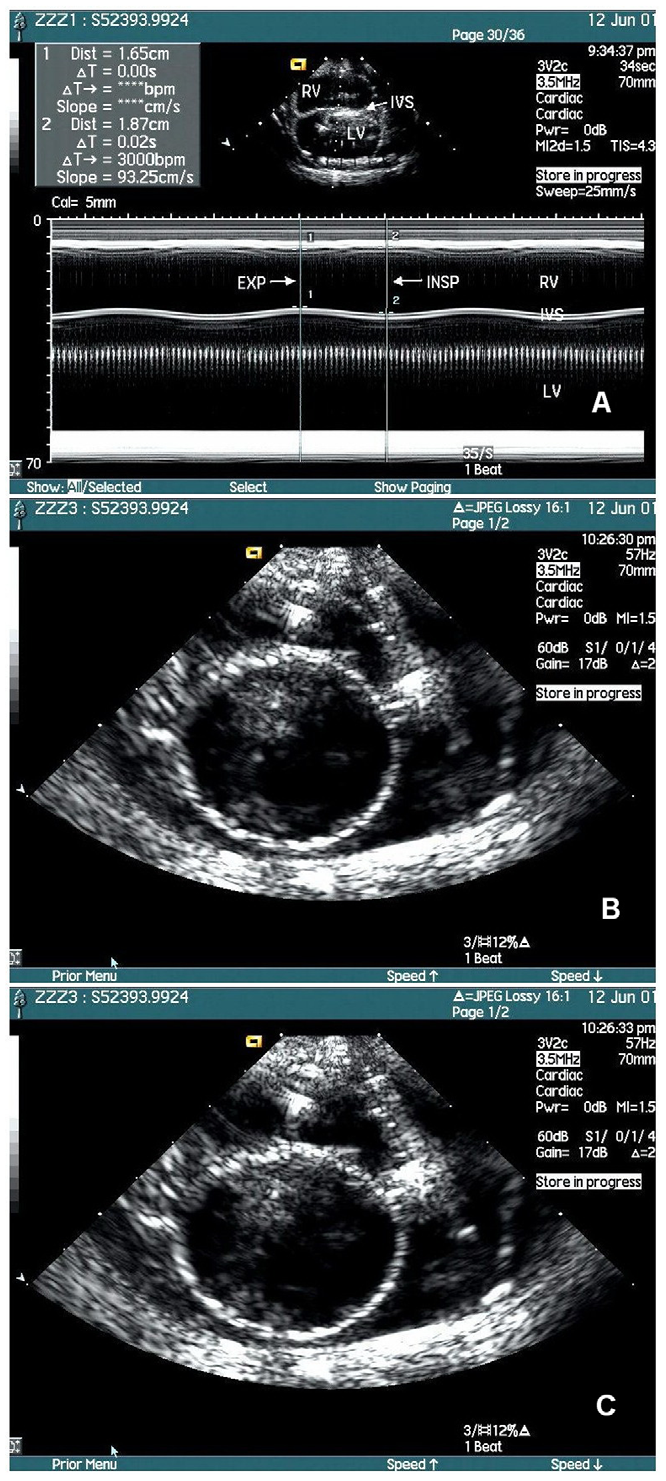
The influence of simulated RIPC on the motion of the simulated IVS. Under the simulated RIPC, the simulated IVS is swinging left- and rightwards (down and up in the figure) to correspond the pressure change in SCC. To simulate the in vivo condition where the right ventricle anterior wall and the left ventricle posterior walls are kept in touch with the chest wall, the pressures in SRV, SLV, SCC and SP are adjusted to 4 mmHg, 9 mmHg, 0 mmHg and 0 mmHg respectively. Acuson’s Sequoia 512 ultrasonographic system was used to record the M-mode and two-dimensional cineloops of the simulated IVS’s motion under the simulated RIPC. Total of 150 frames were captured. **A**. With rhythmic pressure change in SCC, or under the simulated RIPC of 0 to −4mmHg, the IVS swung left- and rightwards at amplitude of 2.2 mm. **B and C**. These two of the total 150 frames demonstrate the 2D echocardiograms of the end-expiration (B) and end-inspiration (C) phases. Their internal LV diameters are 35.0 and 32.8 mm, respectively. RIPC = respiratory intrathoracic pressure change; PVR = pulmonary venous return system; SVR = systemic venous return system, SCC = simulated chest cavity; SRV = simulated right ventricle, SLV = simulated left ventricle; SP = simulated pericardium, IVS: interventricular septum.

In Test 1, with the pressure in the SRV and SLV kept constant (standard pressure condition for model 2), each step of pressure decrease in the SCC from 0 mmHg to −5 mmHg, the simulated IVS moved leftwards (to the SLV direction) in a corresponding position and from 0 mmHg to +5 mmHg, rightwards correspondingly, i.e., it is a corresponding relationship between the pressure in SCC and the position of simulated IVS which we called pressure-position relationship. With rhythmic pressure change in SCC, or under the simulated RIPC of 0 – −4mmHg, the SIVS swung left- and rightwards at amplitude of 2.2 mm (Figure 4).

#### 2.2. Effect of the pressure difference between SRV and SLV on the motion of the simulated IVS

In Test 2, under the same simulated RIPC (0 – −4mmHg), the amplitude of simulated IVS motion increased with the pressure increase in SRV and reached its maximal of this test when the pressure in SRV was equal to that in SLV, 9 mmHg. With the pressures in the two simulated ventricles changing from the standard pressure condition for model 2 to be the pressure equalization, the amplitude of SIVS motion changed from 2.2 mm to 17.6 mm.

#### 2.3. Effect of the pressure in pericardium on the motion of the simulated IVS

In Test 3, with gradual pressure increase in the simulated pericardial space, the SRV collapsed firstly and then the SLV with further pericardial pressure increase to be equal to that in SLV. When the pressure in SRV was increased to be equal to that in SLV, the simulated IVS motion was 22.4 mm in amplitude that was about 10 times more than that in the Test 1 (2.2 mm).

## Discussion

### 1. Previous Studies and Theories about the Mechanism of Pulsus Paradoxus

Previous studies proposed controversial theories regarding the mechanism of pulsus paradoxus though the results of their observations were substantially consistent. Some studies contributed to the mechanism of normal respiratory hemodynamic variations [15], [16], and others focused on the mechanism of pulsus paradoxus in cardiac tamponade [17]–[19]. However, none of the previous studies has fully elucidated the true mechanism.

One of the most common explanations for pulsus paradoxus proposes that an inspiratory increase in right ventricular filling precedes a decrease in left ventricular filling due to the increased pulmonary venous compliance, and that left ventricular stroke volume increases only after two or three cardiac cycles necessary for the increased right heart stroke volume to traverse the pulmonary circulation [4], which was called “lung pooling” hypothesis. As Test 3 of Model 1 in this study showed, the RIPC does not directly influence the distribution of the blood in pulmonary circulation and LV in diastole. The volume increase of pulmonary circulation is the secondary effect of the increased RV filling which is caused by inspiratory, i.e., the increased preload amplify the contraction of RV in systole. The real hydromechanical mechanism of the blood pooling is the inspiratory decrease of LV pressure. Gonzalez et al [9], [20] using high fidelity hemodynamic pressure recordings and Doppler echocardiographic techniques have showed that, in the model of acute cardiac tamponade and in multiple ways, the left and right-sided cardiovascular hemodynamics (ventricular systolic pressure, ejection times and outflow velocity) exhibit an inverse relation during cardiac tamponade that appears to be very close to 180° out of phase. The reciprocal changes in pressure and flow velocity are seen during every respiratory cycle even if preceded by a period of apnea, and the magnitude of change in ventricular pressures and output flow velocity for individual beats is determined by the precise timing of ventricular filling and ejecting to the phase of respiration. If the “lung pooling” hypothesis is true, maximal pulmonary artery pressure and flow in cardiac tamponade would seldom be coincident with minimal aortic pressure and flow, and right and left ventricular stroke volumes would not be 180° out of phase.

An alternative theory about the mechanism of pulsus paradoxus proposes that total pericardial volume is “fixed” in cardiac tamponade, and that augmented filling in one ventricle would result in an immediate and opposite change in the volume of the other ventricle [5]–[7]. Based on this theory, the pulmonary arterial pressure and flow would be nearly reciprocal to the aortic pressure and flow [8]. Settle et al [21] also proposed that the increase in venous return flow during inspiration results in a marked and exaggerated increase in right ventricular dimensions accompanied by a reduction in left ventricular dimensions and flattening and displacement of the septum toward the left ventricle and thus decreases the hemodynamics of the left heart. However, this theory doesn’t reveal the mechanism but a description of the observed phenomenon.

There are also many other studies using various models to simulate pulsus paradoxus and try to disclose its mechanism [22]–[26]. In common with our study, most of these studies have noticed the important role of septal swing in the occurance of pulsus paradoxus. Ramachandran et al [22] used their complex H-CRS model to simulate hemodynamic and respiratory changes associated with tamponade clinically, focusing particularly on the role of the interventricular septum. Their study showed that pulsus paradoxus was a multifactorial phenomenon which is related with septal motion, atrioventricular and right-left ventricular interactions, pulmonary blood pooling, and the depth of respiration. Their model also provided biophysically-based insights helpful for future experimental and clinical study of cardiac tamponade and related pericardial diseases. Kingma et al [24] concluded that the position and shape of the IVS at end-diastole are determined by the transseptal pressure gradient using their animal model, which is consistent with the results of the current study.

### 2. The Main Points of Our Proposed Hypothesis

#### 2.1. Primary influence of RIPC on SVR, PVR and IVS

To help understand the effects of respiratory intrathoracic pressure changes on the systemic venous return, we simplified the complex hydromechanical cardiovascular system by observing only the static pressure component in this system. In Model 1, the stable pressure that simulates the filling pressure of the peripheral venous system was created by employing the gravitational force. The model is hydromechanically equivalent with an enclosed fluid partially in a closed cavity and essentially met the hydromechanical conditions of a real SVR in terms of static pressure component. The peripheral veins of SVR are under the stable atmospheric pressure and continuously receive the blood supply from the capillaries. An inspiratory intrathoracic pressure decrease will decrease the pressure in RV, cause a blood flow increase towards it, and then RV dilates. The increased flow velocity to the RV, the kinetic energy would be subsequently converted to the pressure energy when the flow stops in RV. As a result, the RV pressure would not substantially decrease. Eventually, the inspiratory intrathoracic pressure decrease is theoretically transformed into a RV dilation that is equivalent to a RV filling pressure increase, while the pressure in RV is basically unchanged like Model 1 Test 1 and 2 showed. During expiration, the opposite changes would occur.

Though the real pulmonary-left heart system is much more complex in structure, it could essentially be considered as an enclosed fluid in a closed cavity based on the theory of topology, shown in Model 2. To help understand the complex situation, we propose to exclude the action of gravitational force and to expand the conception of Pascal’s Law in this model. It stated that “A change in pressure applied to an enclosed fluid is transmitted undiminished to every point of the fluid and walls of the containing vessel”. It may be deduced that no matter how complex the structure of the pulmonary vasculature and the lung is, RIPC would obey the law and be transmitted undiminished to every point of the fluid and walls of the containing vessel. In another words, RIPC would be fully transmitted into the whole PVR system without blood redistribution within it as shown in Model 2. This should also be true even when the dynamic feature of the circulation is considered.

From above, we may conclude that the inspiratory intrathoracic pressure decreased causes no substantial pressure change in RV by increasing systemic venous return (SVR), while causes a pressure decrease in LV without blood redistribution in pulmonary venous return (PVR) system. Thus, the ultimate influence of the inspiratory intrathoracic pressure decrease is the generation of a pressure gradient between LV and RV across the IVS.

#### 2.2. Influence of RIPC on the motion of IVS and the affecting factors

As discussed above, the same RIPC has different influence on PVR and SVR, causing a pressure gradient across IVS, which would push IVS towards the left ventricle during inspiration and the right ventricle during expiration. This might be the direct reason for pulsus paradoxus.

There are three factors that would determine the IVS’s motion amplitude as shown in Model 2 tests. As Test 1 in Model 2 demonstrated, the magnitude of the simulated RIPC is the first major factor influencing the simulated IVS motion amplitude. This is reasonable since the simulated RIPC is actually the primary motive force that generates the pressure gradient between the simulated ventricles and moves the simulated IVS. This has also been be verified by an echocardiographic study in human subjects. Though the swing of IVS is hardly noticed in most normal subjects with quiet respiration, it is markedly enhanced in many of them with deeper or resistant respiration [27].

The second important factor that influences IVS motion is the respective pressure in SRV and SLV. When the pressure in SLV is higher than that in SRV, the simulated IVS is tense and the simulated RIPC could only decrease, but not be able to reverse the pressure gradient between the two sides of the simulated IVS, which could also explain why the simulated IVS motion in normal subjects is limited. When the pressure gradient across IVS is small or close to zero, the simulated IVS would relax and the IVS motion would be larger or reach its maximum as test 2 in Model 2 demonstrated.

The third factor that influences the simulated IVS motion amplitude is the simulated intrapericardial pressure. Test 3 in Model 2 demonstrated that with the simulated intrapericardial pressure increase, for example, when the total intrapericardial volume decrease or the heart being compressed, SRV collapsed firstly and, then, SLV. With the pressure in SRV increased to be equal to the pressure in SLV (equilibration) shown in Test 3 in Model 2, the simulated IVS motion was markedly increased. In clinic, multiple factors are usually involved in determining the IVS motion.

### 3. The Potential Application of the Proposed Hypothesis in Clinical Situation

The clinical situation in human body is much more complex. The two muscular pumps intermittently receive and expel the viscous blood to keep it circulate in an elastic tubing system that is nonlinearly pressure-dependent (Starling resistor) [28]. The two have to be separated hydromechanically as they are operating at a very different pressure level. Though the whole body is under the atmospheric pressure and gravitational force, the intrathoracic part of the circulation system still has to suffer from RIPC while the peripheral vasculature is under the stable atmospheric pressure.

The two venous return systems in man,PVR and SVR, are filled and distended by their respective static filling pressures. Because the pressure in the left side of the heart is higher, the shared wall of LV and RV, i.e. IVS, bulges tightly towards the right side. It is evident that the degree of this bulge depends on the pressure difference between the two sides of IVS. As an additive external pressure, RIPC is superimposed on the left side of the original static pressure and modulate it. So it determines the position of IVS and the LV end-diastolic filling volume, thus the output. In addition, the smaller the primary pressure difference between LV and RV is, the easier the IVS swings under a given RIPC.

In normal subjects, as the dilation of the two ventricles are relatively not restricted, the ventricular filling mainly relies on the free-wall distention but not IVS motion. In addition, normally RIPC is small and the static pressure in LV is higher than that in RV. Taken together the motion of IVS is limited and the respiration-driven hemodynamic variation of LV is very small in normal situation [5]–[7].

While in pericardial effusion, the intrapericardial pressure increases. The RV filling might firstly be impaired as normally its filling pressure is relatively lower than that in LV. The human body would enhance RV filling pressure to compensate the life-frightening low RV stroke volume, and lead the increase of the pressure in SVR and RV. This would augment the IVS’s motion amplitude. When the intrapericardial pressure increases to be equal to RV and LV pressures (equilibration) [15], [29]–[31], the IVS motion would reach its maximum (Test 3 in Model 2). The RIPC-driven hemodynamic variations would correspondingly be enlarged. In addition, as the heart is compressed in the condition of pericardial effusion, the dilation of the ventricular free walls are restricted and the filling of each ventricle is more dependent on the IVS swinging, which also enlarge the respiration-driven hemodynamic variations. With further increase of the intrapericardial pressure, the RIPC-driven hemodynamic variation would reach its maximum, permitting only one-sided effective ventricular filling. This might be the reason of the fact that the peripheral arterial pressure may vary over 10 mmHg (up to almost 100 mmHg) though RIPC has only a few mmHg alternations in some patients with cardiac tamponade (amplifier).

This hypothesis may provide a new insight into the respiration-related hemodynamics and help for seeking methods for noninvasive quantification of the intracardiac pressure [27], [32].

The main limitation of this study is that the complex cardiovascular system was simplified from the perspective of mechanics. While the models and tests involved in this study could accurately replicate the pressure relationship among the respective parts of the cardiovascular system. Thus it would not change the conclusions of this study.

### Conclusions

The mechanism of the pulsus paradoxus has been verified using mechanical Models in this study. We found that the anatomical arrangement of the two venous return systems in the thorax leads to the different effects of RIPC on LV and RV and thus a pressure gradient across IVS that tends to swing it left- and rightwards. Three factors that influence the magnitude of IVS have been revealed: RIPC, the respective pressure in SRV and SLV and the intrapericardial pressure. Normally, this swing is limited. When the leftward motion of IVS reaches to a considerable amplitude in some pathologic conditions such as cardiac tamponade, the pulsus paradoxus occurs.
